# How democracies prevail: democratic resilience as a two-stage process

**DOI:** 10.1080/13510347.2021.1891413

**Published:** 2021-04-27

**Authors:** Vanessa A. Boese, Amanda B. Edgell, Sebastian Hellmeier, Seraphine F. Maerz, Staffan I. Lindberg

**Affiliations:** a V-Dem Institute, Department of Political Science, University of Gothenburg, Gothenburg, Sweden; b Department of Political Science, University of Alabama, Tuscaloosa, AL, USA; c WZB Berlin Social Science Center, Berlin, Germany

**Keywords:** Democratic resilience, democratic survival, democratic breakdown, autocratization, judicial constraints

## Abstract

This article introduces a novel conceptualization of democratic resilience - a two-stage process where democracies avoid democratic declines altogether or avert democratic breakdown given that such autocratization is ongoing. Drawing on the Episodes of Regime Transformation (ERT) dataset, we find that democracies have had a high level of resilience to onset of autocratization since 1900. Nevertheless, democratic resilience has become substantially weaker since the end of the Cold War. Fifty-nine episodes of sustained and substantial declines in democratic practices have occurred since 1993, leading to the unprecedented breakdown of 36 democratic regimes. Ominously, we find that once autocratization begins, only one in five democracies manage to avert breakdown. We also analyse which factors are associated with each stage of democratic resilience. The results suggest that democracies are more resilient when strong judicial constraints on the executive are present and democratic institutions were strong in the past. Conversely and adding nuance to the literature, economic development is only associated with resilience to onset of autocratization, not to resilience against breakdown once autocratization has begun.

## Introduction

Democracy is under threat globally. Over 20% of countries in the world^1^ and one-third of the global population are now experiencing substantial and sustained declines in democracy amounting to a “third wave” of autocratization.^2^ Since 1992, 36 democratic regimes have broken down. What distinguishes democracies that prevail against a global wave of autocratization from those that do not?

Understanding “democratic resilience” – the ability to prevent substantial regression in the quality of democratic institutions and practices – is now more important than ever.^3^ Yet, the term presently lacks a clear specification in the literature, making it prone to becoming yet another buzzword in democracy promotion. We offer a new conceptualization of democratic resilience with two stages that are distinct. In the first stage – *onset resilience* – some democracies are resilient by preventing autocratization altogether, meaning they have not experienced substantial or sustained declines in democratic qualities (such as Switzerland and Canada). If onset resilience fails, democracies experience an episode of autocratization. A democracy may then exhibit *breakdown resilience* by avoiding democratic breakdown in the second stage (such as South Korea from 2008–2016, and Benin from 2007–2012).

This two-stage concept of democratic resilience is pragmatic and empirically observable, allowing us to assess which democracies withstand the forces of autocratization (that is, have high onset resilience – at least thus far) and which have breakdown resilience once autocratization has begun.

We make use of the new Episodes of Regime Transformation (ERT) dataset^4^ that identifies episodes of substantial and sustained changes in levels of democracy for most political units from 1900 to 2019 drawing on the V-Dem electoral democracy index (EDI).^5^ This episodes approach enables us to empirically observe the two-stage process of democratic resilience that provides a better concept-measurement validity compared to data on annual changes in levels or discrete regime types.^6^

We then provide a comprehensive overview of global trends in both stages of democratic resilience since 1900. This descriptive analysis offers several new insights. First, it shows that onset resilience is very high among democracies. There have been only 96 episodes of autocratization in 64 democratic countries from 1900 to 2019. Second, however, we find that democracies are increasingly susceptible to onset of autocratization and the period since the end of the Cold War is the worst on record. Third, once a democracy enters an autocratization episode, the fatality rate is distressingly high: since 1900 a mere 19 episodes (23%) managed to avert breakdown at the end of the episode. Fourth, the two-stage approach to democratic resilience demonstrates an important methodological insight: what is typically treated as a quandary of measurement (levels vs. discrete changes) is actually the equifinality of democratic survival.

Finally, we provide a novel set of analyses by modelling how economic and political factors identified as determinants of autocratization in the literature are related to each stage of the democratic resilience process. Judicial constraints on the executive and a country's past experience with democracy (democratic stock) are positively associated with onset and breakdown resilience. Thus, our results support views that see the judiciary as the last bulwark against autocracy. Contrarily, economic development is only associated with resilience to onset of autocratization, not to resilience against breakdown once autocratization has begun. Higher levels of democracy in neighbouring countries, by contrast, are positively related to resilience against breakdown but not to onset resilience. The main takeaway from these empirical correlations is that different factors seem to matter for onset and breakdown resilience, respectively. By adopting an episode approach rather than measuring regime transitions as events, we can distinguish between these factors.

## Conceptualizing democratic resilience

In general, we define democratic resilience as the persistence of democratic institutions and practices. Empirically, resilience is measured as the continuation of democracy, without substantial or sustained declines in its quality, that is, the avoidance of autocratization.^7^ We speak of *episodes* of autocratization to capture periods with a definitive start and end date during which substantial and sustained declines in democratic qualities take place.^8^ Such declines may result in democratic breakdown, or the regime could avert breakdown by reversing the trend and sustain minimal levels of democracy necessary to be considered democratic.

**Figure 1.**
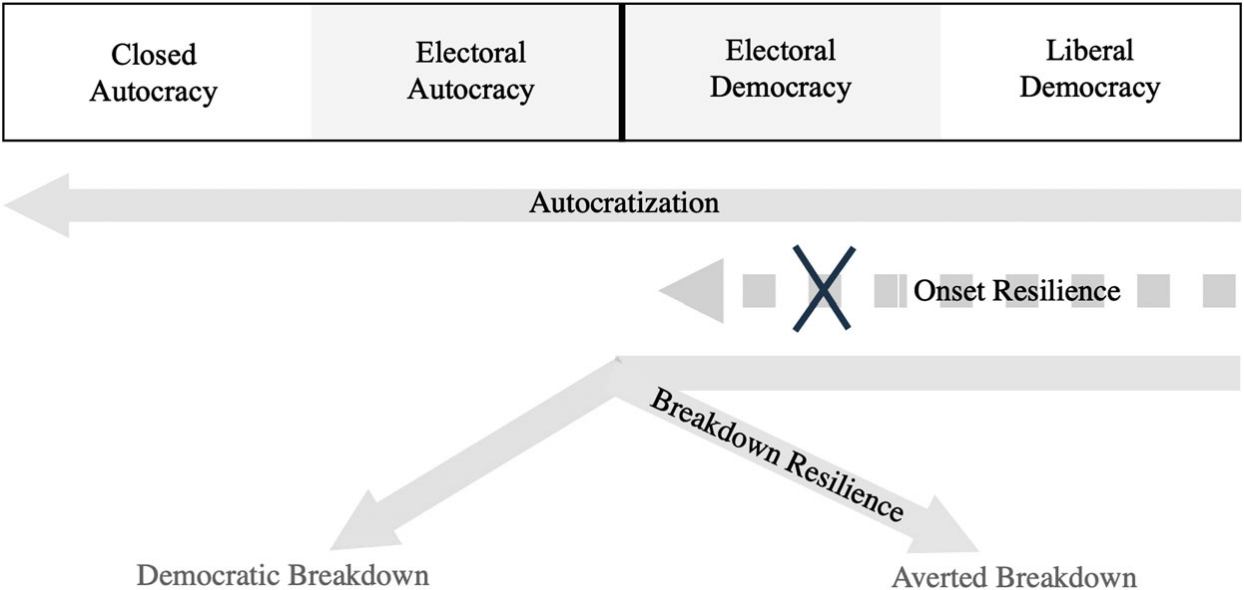
Conceptualization of onset and breakdown resilience.

For this reason, we conceptualize democratic resilience as a two-stage process (see Figure 1). In the first stage, democracies exhibit resilience by maintaining or improving their level of democracy. Put differently, first-stage resilient democracies avoid the onset of autocratization. For this reason, we refer to the first stage as *onset resilience*. In the second stage, democracies that are experiencing autocratization can demonstrate resilience by averting democratic breakdown. This second stage of democratic resilience thus involves avoiding a regime change. We refer to this second stage as *breakdown resilience.* Because a democracy can only exhibit breakdown resilience if it has failed to demonstrate onset resilience, these two stages of resilience may have different drivers. What happens after a democratic breakdown, lies outside the scope of this study.

Importantly, we can only observe whether a democracy has exhibited onset or breakdown resilience *until now*. We may also not yet know if a democracy currently undergoing autocratization will exhibit resilience breakdown because the episode is still ongoing (as is the case for the 12 “censored” episodes in our sample). In either case, this does not necessarily mean that the regime itself will be onset or breakdown resilient in the future.^9^ In other words, our approach avoids making assumptions about resilience at the regime level.

This departs from earlier literature on democratic consolidation, that sought to label democratic regimes as “consolidated” based on predictions about their propensity to survive.^10^ A democracy is typically considered consolidated if it is unlikely to revert to authoritarianism in the future.^11^ Thus, democratic consolidation remains an inherently fuzzy term that relies on causal inferences about democratic stability or survival drawn from observations about the regime duration and its correlates.^12^ Yet, this ignores the fact that all “consolidated democracies” somehow managed to survive from year-to-year before they became consolidated, and that “unconsolidated democracies” often survive for several years (or decades) before ultimately breaking down.^13^ This is why it is preferable to use “resilience” based on empirics rather than the future-orientated and therefore largely unobservable concept of “consolidation”.^14^ We thus provide an important corrective to previous research on democratic consolidation.^15^

If anything, recent failures of onset resilience in cases like the United States, India, and Brazil, as well as failures of breakdown resilience in Hungary and Venezuela, highlight the dangers of forecasting regimes as “consolidated” based on their past. Whether regimes that have previously shown breakdown resilience are more likely to exhibit onset or breakdown resilience in the future is an empirical question yet to be explored. Elsewhere in this special issue, Laebens and Lührmann^16^ provide a detailed qualitative analysis of such breakdown-resilient democracies where autocratization stopped short of a regime transition.

**Figure 2.**
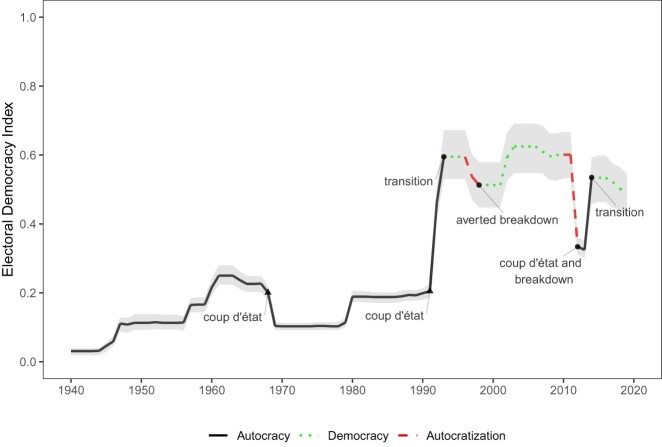
Electoral democracy, coups, and regime transitions in Mali, 1940–2019.

Consider, for example, the case of Mali (Figure 2). Despite Mali's rapid democratization from 1991–1993, early observers warned that it could yield yet another failed democratic experiment.^17^ Prior to 1992, the country was persistently authoritarian, having endured spells of military and one-party rule since independence in 1960. Scepticism about Mali's democratic resilience initially appeared warranted. Widespread irregularities and opposition boycotts marred the 1997 parliamentary elections, forcing the Constitutional Court to invalidate the poll and order a re-run. Against all odds, however, Mali exhibited breakdown resilience and further democratized throughout the mid-1990s and early 2000s. By 2012, it appeared poised for a third peaceful transition of power through multiparty presidential elections. While on the outside Mali had become “one of Africa's model democracies”,^18^ on the inside, its democratic resilience was only as hollow as its institutions. Systemic corruption, strong presidentialism, and weak political parties combined produced rising popular discontent. Meanwhile, the regime's undermining of decentralization spurred a resurgent rebellion in the North. These factors eventually culminated in military coup d’état in March 2012 and a complete failure of democratic resilience.

The fate of Mail's third republic serves as a cautionary tale for those studying democratic resilience or consolidation. In 1997–1998, we see effective breakdown resilience in the face of a political crisis, despite a long history of authoritarian rule. By contrast, after twenty years of democratic elections, the complete failure of democratic resilience (at both stages) in 2012 caught many observers by surprise because they had been fooled by the “consolidation mirage”.^19^

Our conceptualization of democratic resilience also resolves a long-standing discussion in the literature about the relative value of continuous versus categorical regime typologies. We recognize the merits of both approaches, viewing regime characteristics along a continuum from democracy to autocracy (liberal to closed), while also acknowledging the empirical clustering of regimes along this continuum as distinct subtypes. At the most general level, we consider the divide between democracy and autocracy to be a meaningful distinction.

Previous insights on democratic resilience tend to measure regime transitions as events, with survival or durability as the absence of a breakdown in a given year.^20^ This approach overlooks the important conceptual distinction between the avoidance of autocratization altogether and the ability to avert breakdown once autocratization has begun. A rich comparative literature suggests that democratic breakdowns are the culmination of processes of regime transformation producing substantial declines in democracy that often unfold over an extended period,^21^ and do not always culminate in complete democratic breakdown.^22^ Focusing on democratic breakdowns “blind[s] us to potentially important and theoretically revealing cases”.^23^ From a methodological perspective, this also leads to questions about selection bias, especially if factors influencing the experience of autocratization are correlated with the outcome. The other standard quantitative approach is to measure resilience as unchanged year-to-year scores on an index in time-series cross-sectional designs.^24^ Yet, this approach makes it impossible to distinguish democratic decline from breakdown. As such, existing theories about democratic resilience remain incomplete until we simultaneously account for its two stages: onset and breakdown resilience.

## Operationalizing democratic resilience

We make use of the new Episodes of Regime Transformation (ERT) dataset where episodes of autocratization are measured as periods of substantial and sustained declines on the V-Dem electoral democracy index (EDI), which is based on Dahl's conceptualization of polyarchy.^25^ It provides identification of the onset and end dates as well as the outcome of autocratization in democracies (that is, whether democratic breakdown occurred or was averted). The ERT considers substantial and sustained declines (that is, autocratization episode onset) to begin with an annual EDI drop of at least 0.01, followed by an overall decline of at least 0.10 throughout the episode. Autocratization is considered ongoing so long as (I) annual EDI declines continue for at least one out of every five consecutive years, (II) the EDI does not increase by 0.03 or greater in a given year, and (III) the EDI does not gradually increase by 0.10 over a five-year period. The end date of all episodes is the year the case experienced an annual decline of at least 0.01 after episode onset and prior to experiencing one of these three conditions for termination. Breakdown occurs if a country (a) becomes a closed autocracy as defined by the Regimes of the World classification (b) becomes an electoral autocracy for at least one election, or (c) becomes an electoral autocracy for at least five years. Ongoing episodes are censored.^26^

For our purposes here, *onset resilience* is indicated by the absence of an autocratization episode within a given democratic country-year. *Breakdown resilience* is indicated by the absence of a democratic breakdown within an ongoing episode of autocratization. Thus, the ERT allows us to attain a high degree of concept-measure validity when compared to discrete regime type datasets or annual changes on interval democracy measures.

## Democratic resilience over space and time

This section offers a panoramic overview of global trends in democratic resilience from 1900 to 2019. We report on three main findings: First, democracies have been highly resilient to onset of autocratization, but second, this resilience is now substantially weaker in the period after the Cold War. Third, fatality rates are very high once autocratization has started; only slightly more than one in five (23%) regressing democracies avert breakdown. The increasing number of democracies undergoing autocratization, including major G20-countries such as Brazil, India, Indonesia, and the United States, could therefore signal the global democratic tide is turning.

In Table 1, we report statistics for onset resilience before (1900–1992) and after (1993­–2019) the end of the Cold War. We chose these two periods because (a) they reflect changing international norms about liberal democracy and (b) they roughly correspond to the period before and during the present wave of autocratization identified by Lührmann and Lindberg.^27^

**Table 1.** Onset resilience.

	Lack of onset resilience (episodes)		Onset resilience (country-years)		
Period	N onset	% onset	N risk*	N resilient	% resilient
1900–1992	37	39%	2 186	2 149	98%
1993–2019	59	61%	2 188	2 129	97%
Total	96	100%	4 374	4 278	98%

* Risk set includes democratic country-years not in an ongoing episode.

Table 1 demonstrates first that democracies exhibit high onset resilience, avoiding autocratization more than 98% of the time. Out of 4,374 democratic country-years at risk of autocratization (that is, not currently experiencing an episode), 4,278 did not experience episode onset. Put differently, there are only 96 episodes of autocratization affecting 516 democratic country-years in 64 countries from 1900 to 2019.

Second, onset resilience among democracies has deteriorated since the end of the Cold War. From 1900–1992 and 1993–2019, we see fairly similar numbers of democratic country-years at risk of autocratization onset. In the former, democracies showed onset resilience about 98% of the time, as compared to a slight decrease to 97% in the post-Cold War period. However, these numbers obscure a key finding when looking at data from the episode level. We find that 59 (61%) of the autocratization episodes began between 1993 and 2019. This amounts to about 2.27 new autocratization episodes in democracies per year since 1993, as compared to just 0.4 per year in the preceding period. Apart from cases in the 1930s, the failure of onset resilience is overwhelmingly a post-Cold War phenomenon, lending support to arguments that despite (or perhaps because of) a global democratic “zeitgeist”,^28^ democratic resilience is on the decline.

**Figure 3.**
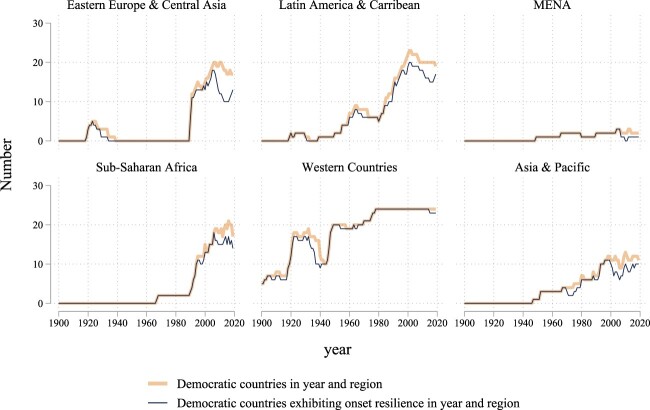
Regional trends in onset resilience from 1900 to 2019. Thick, orange lines depict the total number of democratic countries in each region by year, while the thin, blue lines indicate the number of onset-resilient democratic countries (that is, those not experiencing autocratization in that year).

The decline in onset resilience appears to be irrespective of geopolitical region. Figure 3 plots the number of democratic countries exhibiting onset resilience in a given year (thin, blue lines) against the total number of democracies in that region (thick, orange lines) from 1900 to 2019. The gap between these two lines corresponds to the number of democracies in the region that lacks onset resilience. For most of the regions, we observe the post-Cold War decrease in onset resilience, particularly since the late 1990s. In Eastern Europe and Central Asia (EECA), this is most pronounced, with just 56% of democracies in the region exhibiting onset resilience at its low point in 2007. While onset resilience in EECA has since increased to 76% in 2019, this might be tied to fewer democracies in the region due to breakdowns in Hungary, Serbia, and Ukraine. Asia and the Pacific (AP) and sub-Saharan Africa (SSA) also show faltering onset resilience since the late 1990s. Meanwhile autocratization in Israel and Turkey threaten democratic resilience in the Middle East and North Africa (MENA), where levels of democracy already tend to be quite low. By contrast, for Western countries, where democracy is arguably the oldest and most prevalent, onset resilience remains fairly robust, aside from the United States which began autocratizing in 2016.

Third and finally, breakdown resilience is very low. As shown in Table 2, among the 84 episodes that had ended by December 2019–12 of the 96 are ongoing with unknown outcome – only 19 (23%) exhibited breakdown resilience. In short, once democracies begin autocratizing, their fatality rate is very high. Here we find similar levels of breakdown resilience in the post-1993 period as in the 1900–1992 period (23% and 22%, respectively), but the unknown fates of twelve censored episodes may alter this finding in the future. So far however, two-thirds of the episodes in which breakdown resilience failed, occurred in the period after 1992.^29^

**Table 2.** Breakdown resilience.

	Episodes				Episode-country-year		
Period	Completed	Averted breakdown			Total	Resilient	
	episodes	N	%	Mean duration (years)	years	N	%
1900–1992	37	8	22%	4.92	153	124	81%
1993–2019	47	11	23%	4.60	298	262	88%
total	84	19	23%	4.74	451	386	100%

Completed episodes and mean duration columns exclude 12 censored episodes ongoing in 2019 for which the outcome is unknown. Mean duration is calculated for all episode years occurring in democracies. For episodes that encounter a breakdown and subsequent autocratic regression, non-democratic years after the breakdown are excluded.

**Figure 4.**
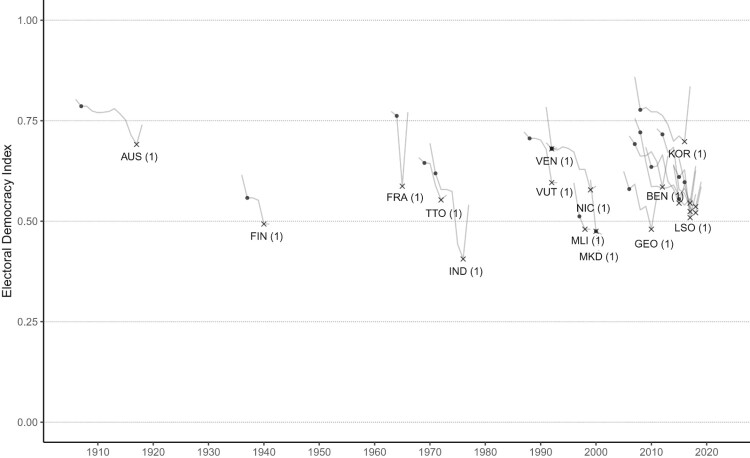
Trajectories of autocratization episodes in democracies that exhibit breakdown resilience throughout the episode, that is, that ended without democratic breakdown. Black dots mark the start year of an episode and the crosses mark the end year. Plots include the pre- and post-episode year. Number of episodes by country in brackets.

**Figure 5.**
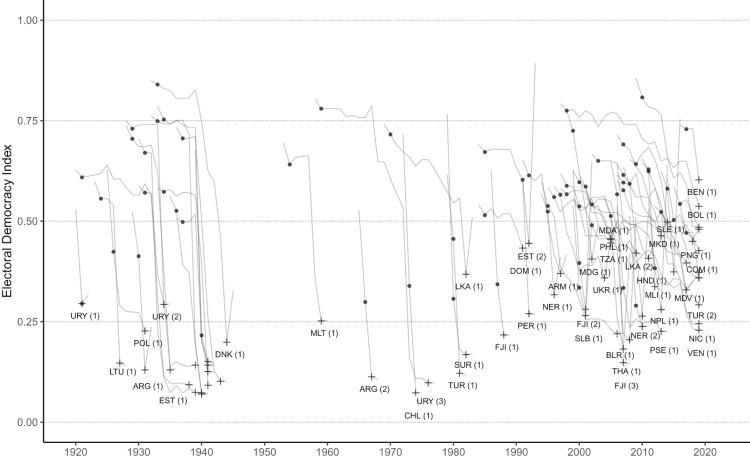
Trajectories of autocratization episodes in democracies that ended with democratic breakdown. Black dots mark the start year of an episode and the crosses mark the end year. Plots include the pre- and post-episode year. Number of episode by country in brackets.

Figures 4 and 5 provide additional detail on the trajectories of democracies undergoing autocratization, divided into those that did and did not exhibit breakdown-resilience, respectively.^30^ A similar plot for censored episodes where the outcome is not known, including present periods for Brazil and the United States, is found in the Appendix (Section B, Figure 7). The clustering of observations in these figures further illustrates the high prevalence of autocratization in post-Cold War period, regardless of outcome. These plots also reveal wide variation in the quality of democracy at the onset of autocratization, in the extent of democratic decline, and the duration of the episode. This demonstrates that taking democratic survival, breakdown, or annual changes at a given point in time would obscure this variation and potentially vital information on patterns that could help us better understand democratic resilience.

When taken together, these findings provide us with the grim observation that democracies have become less resilient in the post-Cold War period. More democracies are experiencing autocratization episodes, and they continue to exhibit a low resilience to breakdown once autocratization has begun. Accordingly, the post-Cold War period has seen the breakdown of an unprecedented 36 democratic regimes. As a result, over 700 million people have lost access to democratic institutions and freedoms.

But what distinguishes the correlates of onset and breakdown resilience, respectively? While we cannot pursue a full-scale explanatory analysis here, in the next section we explore several main factors suggested by the literature.

## Existing insights into determinants of democratic resilience

The literature on democratic breakdown and survival informs much of what we know about resilience. Scholars in this field typically test for the effects of factors on the probability of democratic survival or breakdown as events,^31^ or incrementally using annual changes in levels of democracy.^32^ We suggest a different approach and combine an onset model as well as a selection model following our conceptualization described above, and focus on four of the main factors identified in these two literatures: institutional constraints on the executive, economic factors, neighbouring regimes, and previous democratic experience. We draw on extensive theories in the literature which provide some causal basis to the regressions reported below. In places, we may adopt the causal language which is standard practice in reporting regression models. Nevertheless, acknowledging the limits our analysis due to observational data and statistical techniques, we do not make any firm causal claims here.

### Constraints on the executive

A prominent body of work concerns the “perils of presidentialism”.^33^ According to Linz, separate legislative and executive elections create a dual legitimacy and individual mandate of the executive that predisposes political actors to view presidential systems as a zero-sum game. This discourages coalitions while concentrating substantial powers in one individual.^34^ In effect, presidential systems are more prone to political polarization, deadlock, personalization of politics, and exclusion of losers compared to parliamentary democracies, thus furthering military coups and other types of breakdown.^35^

Noting that the United States is the only long-lasting presidential democracy,^36^ several large-N studies find a negative relationship between presidentialism and rates of democratic survival.^37^ Case evidence suggests that executives in presidential democracies are likely to “rule at the edge of the constitution” because the legislature has limited removal powers.^38^

Recent trends suggest that attacks on democracy are often driven by a concentration of power in the executive, even in parliamentary democracies. This calls for revisiting Linz's focus on the effects of weak constraints on the executive as the chief mechanism linking presidentialism to democratic instability. The extent to which the executive is constrained *de facto* varies considerably, and executive aggrandizement affects both presidential and parliamentary systems.^39^ In effect, the phenomenon of “presidential hegemony” poses a potential risk to democratic resilience across systems.^40^

The Linz thesis is yet to be tested using granular data on the specific causal mechanism of weak constraints on the executive. Our expectation is that stronger constraints on the executive by the legislature and the judiciary are positively associated with both a lower likelihood of autocratization episodes in democracies (onset resilience) and greater resilience to democratic breakdown once such an episode has begun.

### Economic factors

Since Lipset's seminal work on the societal effects of economic development, questions about the links between economics and democratic stability have preoccupied the discipline.^41^ Lipset's original focus is actually on democratic resilience when arguing that “the more the well-to-do a nation, the greater the chances that it will *sustain* democracy” (emphasis added).^42^ Some tests of Lipset's theory such as by Przeworski and Limongi suggest that democracies become resilient to breakdown once they are above a certain threshold level of income.^43^ Several studies find that positive economic growth predicts democratic survival,^44^ but this may be good for the stability of any regime, including autocracies^45^ because a better quality of life makes people more likely to support the status quo over those seeking to undo the existing order.

Indicators of economic development are now standard practice in models estimating democratization, democratic breakdown, and democratic survival.^46^ In line with the bulk of previous studies, we expect that higher levels of economic development will make democracies more resilient to experiencing an autocratization episode (onset). We remain agnostic about the association between development and breakdown resilience.

### Neighbourhood effects

Several studies provide evidence of diffusion effects across countries. This is often described as a “pull towards the regional mean” – or a tendency for countries “left behind” to eventually adapt to regional norms about institutional configurations for autocratic as well as democratic regimes by way of diffusion, emulation, spill-over, or demonstration effects.^47^ In light of the gradual nature of autocratization during the third wave, we expect at most small neighbourhood effects on the probability of experiencing an episode. Once a democracy opts into an episode of autocratization, however, we hypothesize a greater breakdown resilience because dismantling of democracy in stage two should be more difficult for aspiring autocrats in more democratic regions.

### Previous democratic experience

Previous experience under democracy may reinforce democratic resilience through the “construction of solid links between the democratic institutions and society”.^48^ Some scholars suggest that the institutionalization of party systems and judicial institutions^49^ helps to handle “problems of monitoring and social coordination that complicate democratic compromise”.^50^ Others claim that election cycles have a self-reinforcing, self-improving quality, altering the incentives to accept the rules of the game.^51^ Indeed, everyday experiences living under democracy seem to promote democratic attitudes within society, making successful challenges to democracy less likely.^52^ We expect that previous experience with democracy will be associated with a higher onset resilience, as well as with greater resilience to breakdown in stage two.

## Modelling correlates of the two stages of democratic resilience

To estimate onset resilience, we use a standard onset model (probit model with Firth's method of bias reduction)^53^ in which resilient democratic country-years (that is, those not experiencing the beginning of an autocratization episode) are treated as ones. The onset of an episode as given by the ERT is coded as zero and democratic country-years in ongoing episodes are excluded.^54^ To estimate breakdown resilience in stage two, we use a standard bivariate probit model with non-random sample selection.^55^ The first “selection” stage estimates the probability that a given democratic country-year falls within an autocratization episode, that is, it lacks onset resilience, using the sample of democratic country-years in the ERT dataset (estimation sample: 3,864 observations). The second “outcome” stage includes the subsample of country years that are not onset resilient (352 observations in the estimation sample) and estimates the probability of breakdown resilience, thus accounting for selection bias estimated in the first stage. The outcome variable is coded as one for each episode-year in which democratic breakdown does *not* occur and zero for breakdown years.

We focus on factors from the literature discussed above. To capture the key mechanism in the “perils of presidentialism”, we include two *de facto* measures of executive constraints provided by the V-Dem dataset: the judicial constraints on the executive index and the legislative constraints on the executive index.^56^ The former measures judicial independence and whether the executive respects court rulings and the constitution. The latter indicates the degree to which the legislature and government agencies exercise oversight of the executive.^57^ Second, we include measures of inflation-adjusted GDP per capita and economic growth from the Maddison project^58^ to capture the level of economic development and economic performance, respectively. Third, to address spatial clustering of regimes and potential neighbourhood effects found in the literature, we include the average scores of V-Dem's EDI for all other countries in the region using the tenfold geo-political classification scheme in V-Dem.^59^ Fourth, to capture past democratic experience, we draw on a recently developed measure of democratic stock.^60^ Finally, we add a nonlinear time trend to control for unobserved factors that changed over time.

In addition, we include a series of other well-known correlates of democratic resilience. Because military coups are one of the main threats to democracy,^61^ we use information on the occurrence of one or more military coups in a country (binary indicator) by combining information from two coup datasets.^62^ We include population size from the Maddison project^63^ as it might affect a polity's susceptibility to conflict and autocratization. We also count the cumulative number of previous episodes of autocratization in democracies. A large number of previous episodes should be indicative of a general vulnerability to autocratization. To account for global trends, we add the percentage of countries with ongoing democratization and autocratization episodes for each year. We include region dummies to control for unobserved time-invariant factors. Finally, a linear time trend accounts for global trends in autocratization and decade dummies account for global shocks such as the two World Wars simultaneously affecting a large number of countries. Due to missing economic and population data, we exclude 14 episodes in the ERT from our analysis.^64^ We provide summary statistics for all variables in the different samples used in the analysis in Table 8, 9 and 10 in Section E of the Appendix. To reduce concerns of simultaneity bias, that could arise if aspiring autocrats dismantle institutional checks and balances, we lag all variables (except for coups) by one year.

## Results

The main results are summarized in Table 3. Model 1 identifies factors associated with higher levels of onset resilience. In line with scholarly work on the importance of judges and courts for democracy, we find that stronger judicial constraints on the executive are significantly associated with greater democratic resilience to experiencing autocratization. We do not observe a similar relationship for legislative constraints. In line with the literature, we find that economic development and a greater democratic stock are also associated with significantly higher onset resilience. Furthermore, Model 1 shows that coups, previous episodes of autocratization, and a larger population may significantly decrease the likelihood of onset resilience. The significant coefficient for the share of democratizing countries suggests that global trends in democratization has a positive association with onset resilience in individual countries.

**Table 3.** Main results: correlates of onset and breakdown resilience.

	Model 1	Model 2	
	Onset resilience	[I]	Breakdown resilience
Judicial constraints on executive	1*.*52^∗∗^ (0.54)	−2*.*51^∗∗∗^ (0*.*75)	1*.*89^∗∗^ (0*.*70)
Legislative constraints on executive	0*.*08 (0*.*38)	−1*.*43^∗∗∗^ (0*.*51)	−0*.*05 (0*.*48)
GDP per capita (log)	0.33^†^ (0*.*20)	−0.95^∗∗∗^ (0*.*23)	0*.*05 (0*.*32)
GDP growth (5-year avg.)	0*.*00 (0*.*01)	−0*.*04^∗^ (0*.*02)	0*.*02 (0*.*02)
Regional democracy levels	−0*.*02 (0*.*87)	−0*.*16 (1*.*33)	4*.*77^†^ (2*.*45)
Democratic stock	5*.*45^∗∗∗^ * *(1*.*04)	1*.*76^†^ (0*.*99)	3*.*33^∗∗^ (1*.*20)
Coup	−1*.*48^∗∗∗^ * *(0*.*27)	1*.*54^∗∗∗^ (0*.*31)	−2*.*47^∗∗∗^ (0*.*52)
Population (log)	−0*.*05 (0*.*06)	0*.*03 (0*.*08)	0*.*10 (0*.*10)
Previous autocratization episode	−1*.*23^∗∗∗^ (0*.*14)	1*.*30^∗∗∗^ (0*.*16)	* *
Autocratizing countries (%, global)	0*.*02 (0*.*01)	−0*.*01 (0*.*02)	
Democratizing countries (%, global)	0*.*02^∗^ (0*.*01)	−0*.*04^∗∗∗^ (0*.*01)	* *
Episode duration			−0*.*20^∗∗∗^ (0*.*06)
Episode duration^2^			0*.*01^∗^ (0*.*00)
Intercept	−4*.*54^∗∗^ (1*.*72)	10*.*04^∗∗∗^ (1*.*78)	−4*.*08 (3*.*90)
*Ρ*	*−*	0*.*03	
Region dummies	yes	yes	
Nonlinear time trend	yes	yes	
AIC	522.04	1526.38	
BIC	679.05	1805.63	
Log Likelihood	−236.02	−719.19	
Total obs.	3,946	4,216	
Censored obs.	*−*	3,864	
Obs. in outcome stage	*−*	352	

Note: Probit model with Firth's bias reduction (Model 1) and Heckman-style selection model (Model 2). Dependent variable in selection equation [I]: ongoing autocratization in a democracy. Dependent variable in outcome equation: breakdown resilience in current episode-year. Standard errors clustered at the country-level. Time since last autocratization episode (t, t^2^, t^3^) omitted from Table. Significance levels ^∗∗∗^*p *< 0*.*001, ^∗∗^*p *< * *0*.*01, ^∗^*p *< 0*.*05, ^†^*p *< 0*.*1.

Model 2 contains the results from the two-stage Heckman model that we use to assess factors associated with breakdown resilience. The model takes into account that only countries that were not onset resilient can display breakdown resilience. As required by the model assumptions, we include some predictors for “selection into” autocratization in the first model stage but not in the second stage. We argue that the number of previous autocratization episodes and a greater number of concurrent episodes of regime transformations (democratization and autocratization) in other democracies should be expected to influence whether a democracy is more likely to lose onset resilience but that they should be substantively unrelated to the outcome once an episode is ongoing. We also control for the duration of the episode in the second stage, by including the number of years since episode onset and its square term, as shorter or longer episodes may be more prone to breakdown.^65^

The right column of Model 2 shows the results for breakdown resilience in the second stage. Similar to the onset model, judicial constraints on the executive are associated with significant increases in the likelihood of resilience to breakdown. However, legislative constraints are not significant at conventional thresholds (p>0.10). This finding supports recent work claiming that judicial institutions can act as the “last bulwark” against democratic breakdown^66^, while the legislature can do little to stop autocratization once it has started.

The results for economic factors are less clear across the two stages of autocratization. Economic development is significantly associated with onset resilience but not with breakdown resilience. These results could suggest that long-term economic development could make people less inclined to support actors inclined to derail democracy but once autocratization has started, economic factors are less relevant.

Higher levels of democratic stock are significantly associated with increases in resilience to both onset and breakdown. More democratic neighbours are significantly related to higher resilience against breakdown. These results illustrate that factors the literature suggest as causally related to survival and breakdown may have varying relationships to the two stages of resilience. Although we cannot compare both models directly, they suggest that a different set of explanatory factors for each stage of resilience is needed to understand variation in levels of democratic resilience.

**Figure 6.**
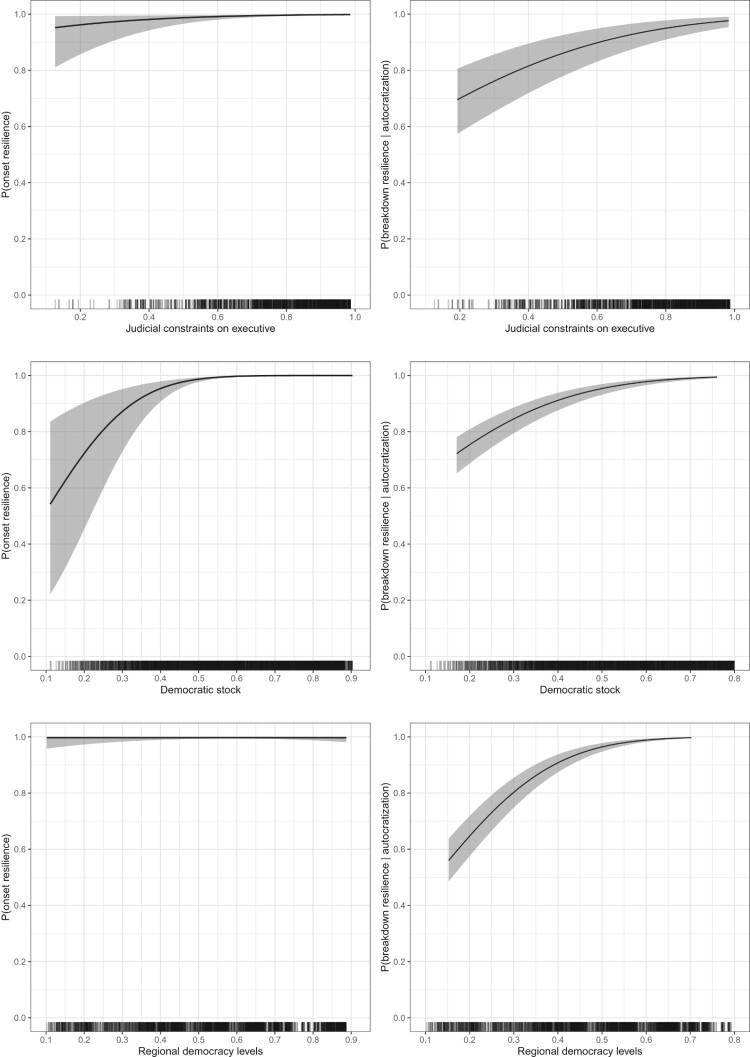
Predicted probabilities of onset resilience (left panel) and breakdown resilience (right panel) over the range of selected explanatory variables. Estimates and 95% confidence intervals are based on simulations from the model parameters.

To illustrate the results more substantively, we simulate predicted probabilities for both stages of resilience based on our model estimates and plot them over the range of the key independent variables in Figure 6. The plots on the left show how the probability of onset resilience varies with judicial constraints on the executive, democratic stock, and regional levels of democracy. Country-years where the de facto constraints on the executive are greater have a higher likelihood of onset resilience. However, the differences are relatively small. Onset resilience is high even at moderate levels of judicial constraints.

The relationship between democratic stock and onset resilience is more pronounced. Democracies with a short history of democracy like Tunisia (0.38 in 2019) or Nepal (0.39 in 2019) face a considerable risk of experiencing autocratization whereas countries with longstanding democratic institutions are highly resilient. Given the rarity of autocratization onset and democratic breakdown, these differences are quite substantial. For regional levels of democracy, the plots show little differences in onset resilience. A comparison of the plots on the left and right underlines the importance of separating onset- and breakdown resilience. In the second stage, judicial constraints are clearly related to a higher likelihood of breakdown resilience. The predicted probability of resilience against breakdown is much lower for countries with low-to-medium levels of judicial constraints. While the effect of democratic stock is comparable to the onset stage, regional levels of democracies only make a difference for breakdown resilience. Thus, different factors matter for the different stages of democratic resilience.

A series of robustness tests give substantially unchanged results (see Appendix, section E Robustness Checks for details). For instance, we run different model specifications, decompose the legislative and judicial constraints indices and apply different thresholds for the starting and end dates of autocratization episodes. A more general challenge is the small number of episodes and the rarity of autocratization onset and democratic breakdown. Thus, including a large number of explanatory variables can be problematic, as can the exclusion or inclusion of influential episode cases. However, our main findings are robust to different modelling choices and operationalization of autocratization episodes. Across models, judicial constraints and democratic stock are significantly associated with both types of resilience. The results for economic factors are not consistent across stages; economic development matters only for onset resilience. Regional levels of democracy appear to be relevant only when autocratization is already ongoing. Coups are associated with lower levels of resilience in both stages.

## Conclusions

Existing quantitative studies of democratic resilience typically operationalize democratic breakdown as events. This disregards conceptual and empirical differences between those democracies that never experience autocratization and those that – having begun autocratizing – somehow manage to avert breakdown. This article conceptualizes and analyses democratic resilience as a two-stage process – either by avoiding the onset of autocratization altogether or, once it has started by avoiding a full breakdown.

With this novel conceptualization and using the ERT dataset, this article offers a first panoramic overview of global trends in democratic resilience from 1900 to 2019. We show that overall, resilience to the onset of autocratization among democracies is high. There have been only 96 cases of episodes of autocratization in 70 democratic countries over the 120 years of available data. Democracies are resilient over 90% of the time.

Second, we also demonstrate that democratic resilience to onset has gone down markedly, and autocratization in democracies is overwhelmingly a post-Cold War phenomenon. Of all the episodes, 59 (61%) began after 1992.

Third, we show that once democracies “select in” to autocratization, breakdown resilience is weaker. The global fatality rate is 77% – only 19 of the 84 completed episodes managed to change the course and avert breakdown. Thus far, the third wave of autocratization has led to the breakdown of 36 democratic regimes, with only eleven cases showing resilience to breakdown. In sum, during the present period democracies are less resilient to onset than before, while the fatality rate remains very high once a democracy experiences autocratization.

In the second part of the article, we examine how some of the prime suspects or covariates from the literatures on democratic survival/breakdown relate to a two-stage understanding of democratic resilience. Modelling both onset and breakdown resilience, we analyse the correlates of resilience. We find corroboration for claims that view the judiciary as the “last bulwark” against democratic breakdown. Judicial constraints are positively and significantly associated with resilience to the onset of autocratization and to democratic breakdown once autocratization has begun. As also discussed elsewhere in this special issue, judicial institutions seem to play an important role as democracy's last line of defence against aspiring dictators. Our results also point to the importance of a country's past experience with democracy, which is consistently associated with higher levels of resilience in both stages.

In a contribution to the literature on the role of economic development for endurance of democracy, we can also nuance the picture. We find that higher level of economic development is associated with a greater onset resilience but has zero influence on avoiding breakdown once an episode has begun. This is an important corrective to what we know from the previous literature that did not distinguish between the two forms of resilience. For breakdown resilience, what seems to matter more is having democratic neighbours and longer previous democratic experiences. This means that our existing theories remain incomplete until we account for the two-stage nature of democratic resilience.

This study further underscores the need for more nuanced research on the role of factors – endogenous or exogenous^67^ – in different stages of democratic resilience. For practitioners in democracy promotion and pro-democracy activists, the spectre of autocratization requires different responses depending on whether the process has already begun. Only then can democracies prevail.

## Supplementary Material

Appendix_color.pdf

## Notes

1 We use “countries” for ease while acknowledging V-Dem's data include semi-autonomous units and colonies in the dataset.

2 Maerz et al., “State of the world 2019”; Lührmann and Lindberg, “A Third Wave of Autocratization.”

3 This builds on concepts of democratic durability and resilience as defined by Burnell and Calvert, “The Resilience of Democracy.”

4 Edgell et al., “Episodes of Regime Transformation.” The attached ERT Codebook provides more information on content and scope of the ERT dataset.

5 Coppedge et al., “V-Dem Dataset V10.”

6 Svolik, “Authoritarian Reversals”; Bernhard, Nordstrom, and Reenock, “Economic Performance”; Alemán and Yang, “A Duration Analysis.”

7 In many cases, this means democratic stability, but it also includes regimes undergoing democratic deepening, or reforms to institutions and practices resulting in even higher levels of democracy.

8 Edgell et al., “Episodes of Regime Transformation”; Lührmann and Lindberg, “A Third Wave of Autocratization”; Wilson et al., “Successful and Failed Episodes.”

9 More technically democratic resilience (*r*) is divided into *onset resilience* (*r^1^*) and *breakdown resilience* (*r^2^*). In a given year (*i*), a democracy (*j*) is either onset resilient ($r_{i,j}^{1}$ = 1) or experiences autocratization ($r_{i,j}^{1}$=0). We observe whether a democracy has exhibited onset resilience *until now* ($min[r_{1,j}^{1}\ldots r_{k,j}^{1}]$ = 1). Likewise, in a given year, democracies experiencing autocratization are either breakdown resilient ($r_{i,j}^{2}$=1) or experience a transition to autocracy ($r_{i,j}^{2}$=0). We know the outcome of the *episode* of autocratization for 84 episodes in our sample in terms of breakdown resilience (i.e. $r_{i,e}^{2}$=1) or not (i.e. they resulted in breakdown, $r_{i,e}^{2}$ = 0).

10 The literature on democratic consolidation discusses various dimensions of consolidation, e.g. behavioural aspects (Foa and Mounck, “The Danger of Deconsolidation” or Chull Shin, “Democratic Deconsolidation in East Asia”) or institutional factors (Merkel, “Embedded and Defective Democracies”). For a discussion of the issues of consolidation as a future-orientated concept, see Schedler, “What Is Democratic Consolidation?”, 91, or Schedler, “Measuring Democratic Consolidation.”

11 Houle, “Inequality and Democracy”; Svolik, “Authoritarian Reversals”; Svolik, “Which Democracies Will Last?”.

12 Schedler, “What Is Democratic Consolidation?”, 91, or Schedler, “Measuring Democratic Consolidation”.

13 Svolik, “Authoritarian Reversals” for a related discussion.

14 For further criticism on the concept consolidation see O’Donnell, “Illusions About Consolidation” and Svolik, “Authoritarian Reversals.”

15 Svolik, “Authoritarian Reversals”; Gasiorowski and Power, “The Structural Determinants”.

16 Laebens and Lührmann, “What Stops Erosion?”.

17 Vengroff, “Governance and the Transition to Democracy.”

18 Wing, “Mali: Politics of Crisis.”

19 Greene, “Coups and the Consolidation Mirage.”

20 ^.^Alemán and Yang, “A Duration Analysis”; Bernhard, Nordstrom, and Reenock, “Economic Performance”; Gasiorowski, “Economic Crisis”; Gates et al., “Institutional Inconsistency.”

21 See Cassani and Tomini, “Reversing Regimes and Concepts”, 18. Levitsky and Ziblatt, “How Democracies Die.”

22 See Przeworski, *Crises of Democracy* for a discussion of two cases of averted democratic breakdown: France during the 1950s and 1960s and the US under Nixon.

23 Ziblatt, “How Did Europe Democratize?” 326.

24 E.g. Teorell, *Determinants of Democratization*.

25 Dahl, *Polyarchy*; Teorell, Lindberg and Skaaning, “Measuring Polyarchy.”

26 Edgell et al., “Episodes of Regime Transformation”. For more technical details on the ERT, see the attached ERT codebook. The authors of the ERT provide an R-package that replicates all episodes based on the most recent V-Dem dataset. The package allows for further robustness tests and has flexible parameter settings to redefine the episode data: Maerz et al., “ERT – an R package.”

27 Lührmann and Lindberg, “A Third Wave of Autocratization”.

28 Linz and Stepan, *Problems of Democratic Transition*.

29 Section C of the Appendix lists all episodes by outcome.

30 The supplemental material includes additional interactive plots that allows the reader to take a closer look at individual country trajectories.

31 E.g. Svolik, “Authoritarian Reversals”; Hollyer, Rosendorff, and Vreeland, “Transparency, Protest”; Bernhard, Nordstrom, and Reenock, “Economic Performance”.

32 E.g. Ginsburg and Huq, *How to Save*; Erdmann, “Transition from Democracy”; Mechkova, Lührmann, and Lindberg, “How Much Democratic Backsliding?”

33 Linz, “The Perils of Presidentialism.”

34 Ibid; Linz, *The Breakdown of Democratic Regimes*; Linz and Valenzuela, *The Failure of Presidential Democracy*.

35 Kaufman and Haggard, “Democratic Decline.”

36 E.g. Cheibub, *Presidentialism, Parliamentarism, and Democracy*.”

37 Mainwaring, “Presidentialism, Multipartism, and Democracy”; Bernhard, Nordstrom, and Reenock, “Economic performance”; Riggs, “Presidentialism versus Parliamentarism”; Svolik, “Which Democracies Will Last?”.

38 Stepan and Skach, “Constitutional Frameworks”. However, several critiques contend that the relationship is spurious, e.g. Cheibub, *Presidentialism, Parliamentarism, and Democracy*; Hiroi and Omori, “Perils of Parliamentarism?”

39 Bermeo, “On Democratic Backsliding.”

40 Pérez-Liñán, Schmidt, and Vairo, “Presidential Hegemony.”

41 Lipset, *Some Social Requisites*.

42 Ibid., 75.

43 Przeworski and Limongi, “Modernization: Theories and Facts”; also see Boix and Stokes, “Endogenous Democratization”; Burkhart and Lewis-Beck, “Comparative Democracy”; Epstein et al., “Democratic Transitions”; Przeworski et al., *Democracy and Development*; Teorell, *Determinants of Democratization*.

44 Gasiorowski, “Economic Crisis”; Gates et al., “Institutional Inconsistency”; Morlino and Quaranta, “What Is the Impact.”

45 Alemán and Yang, “A Duration Analysis”; Feng, “Democracy, Political Stability and Economic Growth”; Gates et al., “Institutional Inconsistency”; Svolik, “Authoritarian Reversals.”

46 Morlino and Quaranta, “What Is the Impact”; Olson, “Dictatorship, Democracy, and Development”; Teorell, *Determinants of Democratization*.

47 E.g. Bader, Grävingholt, and Kästner, “Would Autocracies Promote Autocracy?”; Brinks and Coppedge, “Diffusion Is No Illusion”; Gates et al., “Institutional Inconsistency”; Gleditsch, *All International Politics Is Local;* Gleditsch and Ward, “Diffusion”; Houle and Kayser, “The Two-step Model”; Risse and Babayan, “Democracy Promotion”; Tansey, Koehler, and Schmotz, “Ties to the Rest.”

48 Tomini and Wagemann, “Varieties of Contemporary Democratic Breakdown and Regression”, 690.

49 Pérez-Liñán and Mainwaring, “Regime Legacies.”

50 Reenock, Staton, and Radean, “Legal Institutions and Democratic Survival,” 491.

51 Lindberg, *Democracy and Elections in Africa*, 144.

52 Grosfeld and Zhuravskaya, “Cultural vs. Economic Legacies”; Guiso, Sapienza, and Zingales, “Long-term Persistence”; Persson and Tabellini, “Democratic Capital”; Putnam, *Making Democracy Work*.

53 Kosmidis and Firth, “Jeffreys-prior penalty”; McGrath, “Estimating Onsets.”

54 Due to listwise deletion, we end up with 82 onset cases in our estimation sample.

55 Heckman, “Sample Selection Bias”; Marra and Radice, “GJRM.”

56 Linz, “The Perils of Presidentialism.”

57 While both indices are part of the Liberal Component Index, there is no overlap with the EDI that is used to determine the start and end of autocratization episodes. For the full list of all variables included in the two indices, see Coppedge et al., “V-Dem Codebook v10”, 357.

58 Bold et al., “Maddison Style Estimates”. We use a five-year moving average of GDP growth to make sure our results are not driven by short-term fluctuations. We also use linear interpolation to replace missing values in the data.

59 Brinks and Coppedge, “Diffusion Is No Illusion.”

60 Edgell et al., “Democratic Legacies.”

61 Marinov and Goemans, “Coups and Democracy.”

62 Powell and Thyne, “Global Instances”; Przeworski et al., Political Institutions and Political Events.

63 Bold et al., “Maddison Style Estimates.”

64 A full list can be found in Table 7 in Section A of the Appendix.

65 The second stage is similar to a regular duration model, which is why we add a variable for the duration of the ongoing episode as well as its squared term.

66 Staton et al., *Can Courts be Bulwarks*?

67 For a conceptual differentiation between endogenous and exogenous factors see Gerschewski, “Erosion or Decay?”
