# Supplementary material for: How democracies prevail: democratic resilience as a two-stage process
Source: Democratization. 2021 Apr 27;28(5):885–907. doi: 10.1080/13510347.2021.1891413 (PMC8336576; doi:10.1080/13510347.2021.1891413)
Supplement: Appendix_color.pdf [file FDEM_A_1891413_SM8489.pdf]

Appendix to
"How Democracies Prevail"

A List of Democratic Regression Episodes by Outcome

Table 4. Averted democratic breakdown, 1900–2019

Country	Begin	End
Australia	1907	1917
Benin	2007	2012
Bosnia and Herzegovina	2010	2015
Dominican Republic	2015	2018
Ecuador	2008	2017
Finland	1937	1940
France	1964	1965
Georgia	2006	2010
India	1971	1976
Kosovo	2016	2017
Lesotho	2015	2017
Mali	1997	1998
Moldova	2012	2018
Nicaragua	1992	1999
North Macedonia	2000	2000
South Korea	2008	2016
Trinidad and Tobago	1969	1972
Vanuatu	1988	1992
Venezuela	1992	1992

Table 5. Democratic breakdown, 1900–2019

Country	Begin	End
Argentina	1930	1930
Argentina	1966	1966
Armenia	1992	1996
Austria	1931	1933
Bangladesh	2002	2002
Belarus	1995	1997
Belgium	1937	1940
Benin	2017	2019

Continued on next page

Table 5 – continued from previous page

Country	Begin	End
Bolivia	1998	2019
Burkina Faso	2014	2015
Chile	1973	1973
Comoros	2015	2015
Czech Republic	1934	1939
Denmark	1933	1943
Dominican Republic	1985	1990
Estonia	1929	1934
Estonia	1991	1992
Fiji	1987	1987
Fiji	2000	2000
Fiji	2006	2007
France	1934	1940
Germany	1921	1933
Honduras	1998	2008
Hungary	2006	2018
Latvia	1929	1934
Lithuania	1924	1927
Luxembourg	1940	1940
Madagascar	1996	2001
Madagascar	2009	2009
Maldives	2011	2014
Mali	2011	2012
Malta	1954	1958
Moldova	2000	2005
Nepal	2012	2012
Netherlands	1933	1940
Nicaragua	2003	2007
Niger	1995	1996
Niger	2007	2009
North Macedonia	2007	2013
Norway	1937	1941
Palestine/West Bank	2007	2007
Papua New Guinea	2002	2011
Peru	1985	1992
Philippines	2001	2004
Philippines	2016	2019

Continued on next page

Table 5 – continued from previous page

Country	Begin	End
Poland	1926	1926
Serbia	2004	2015
Sierra Leone	2007	2012
Solomon Islands	2000	2000
Spain	1936	1937
Sri Lanka	1970	1982
Sri Lanka	1998	2006
Suriname	1980	1980
Tanzania	2000	2001
Thailand	2005	2006
Togo	2017	2017
Turkey	1980	1980
Turkey	2007	2014
Ukraine	1997	1998
Ukraine	2008	2012
Uruguay	1921	1921
Uruguay	1931	1933
Uruguay	1959	1973
Venezuela	1999	2003
Zambia	2013	2014

Table 6. Censored democratic regression, 1900–2019

Country	Begin	End
Brazil	2012	2019
Bulgaria	2002	2019
Chile	2011	2019
Croatia	2011	2019
Czech Republic	2009	2019
India	2002	2019
Israel	2010	2019
Niger	2013	2019
Poland	2013	2019
South Africa	2009	2019
Tanzania	2015	2019
United States of America	2015	2019

Table 7. Episodes not included in the analysis due to missing data

Country	Begin	End
Suriname	1980	1982
Kosovo	2016	2017
Latvia	1929	1939
Maldives	2011	2017
Papua New Guinea	2002	2018
Palestinian territories	2007	2013
Estonia	1929	1935
Fiji	1987	1988
Fiji	2000	2001
Fiji	2006	2007
Lithuania	1924	1927
Luxembourg	1940	1941
Solomon Islands	2000	2001
Vanuatu	1988	1992

B EDI trajectories of censored democratic regression episodes

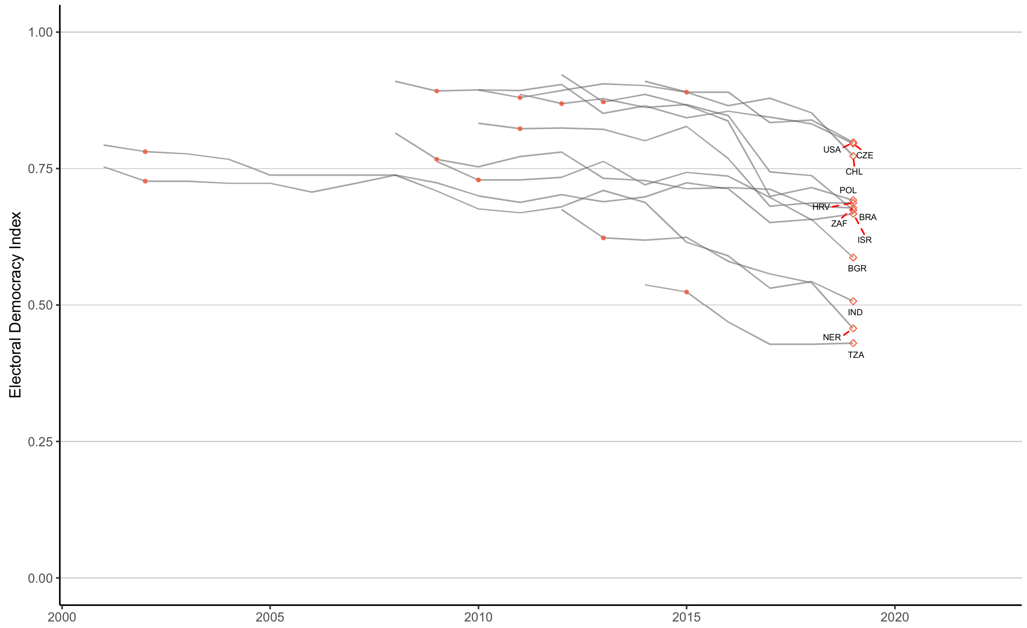

Figure 7. Electoral Democracy Index (EDI) trajectories of countries in ongoing (censored) democratic regression episodes. Red dots mark the start year of an episode. Plots include the pre-episode year.

C Onset resilience by region

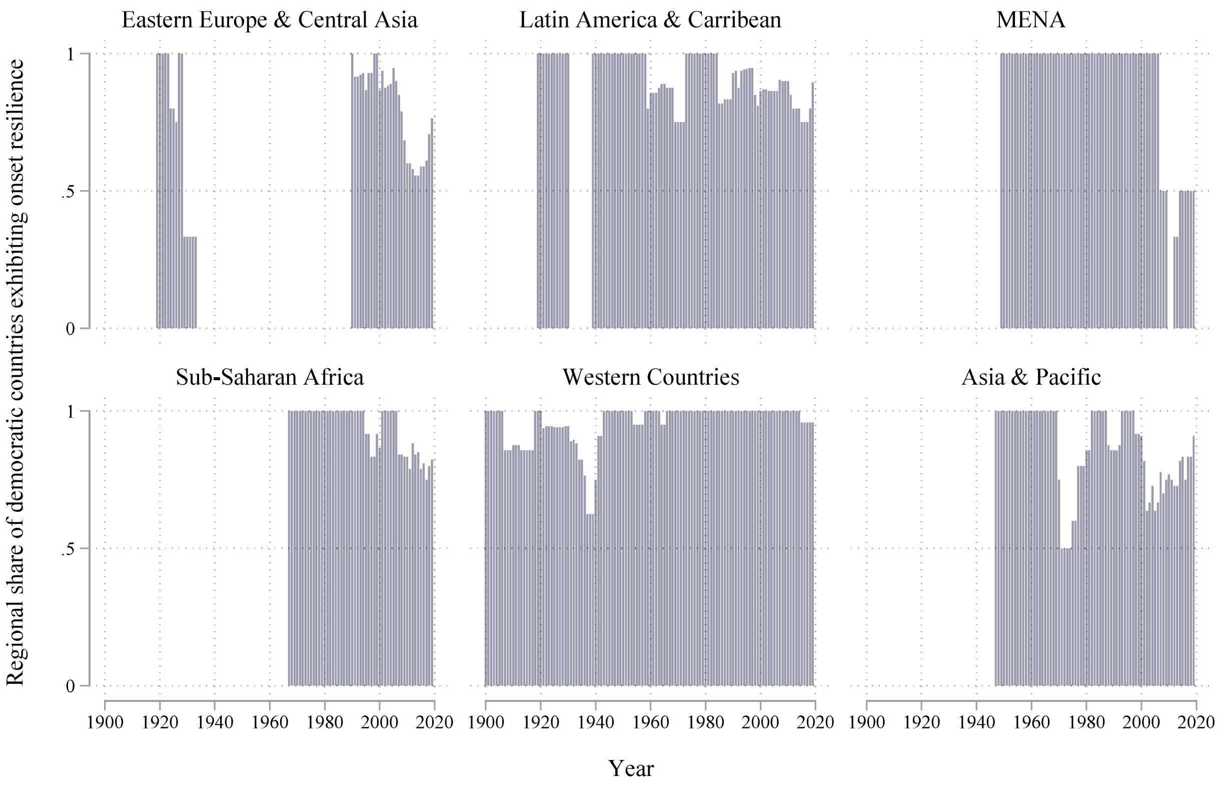

Figure 8. Regional shares of regimes that exhibit onset resilience in a given year from 1900 to 2019.

D Summary statistics for main variables

Table 8. Onset sample including all non-episode years and the first episode year (n = 3,946 country-years)

	Mean	St. Dev.	Min	Max
Autocratization episode	0.02	0.14	0	1
Judicial constraints on executive	0.84	0.14	0.06	0.99
Legislative constraints on executive	0.80	0.15	0.05	0.98
GDP per capita (log)	9.30	0.92	6.50	11.35
GDP growth (5 year avg.)	2.26	3.20	−27.26	75.14
Regional democracy levels	0.59	0.21	0.10	0.89
Democratic stock	0.60	0.19	0.08	0.90
Previous autocratization episodes	0.43	0.62	0	3
Coup	0.01	0.07	0	1
Population (log)	8.93	1.69	4.52	13.84
Autocratizing countries (%, global)	9.42	6.49	0.00	25.71
Democratizing countries (%, global)	19.40	8.46	0.00	41.76

Table 9. Full sample including all non-episode years and all episode years (n = 4,216 country-years)

	Mean	St. Dev.	Min	Max
Autocratization episode	0.08	0.28	0	1
Judicial constraints on executive	0.83	0.15	0.06	0.99
Legislative constraints on executive	0.79	0.16	0.05	0.98
GDP per capita (log)	9.26	0.92	6.50	11.35
GDP growth (5 year avg.)	2.18	3.26	−27.26	75.14
Regional democracy levels	0.58	0.21	0.10	0.89
Democratic stock	0.59	0.19	0.08	0.90
Previous autocratization episodes	0.48	0.65	0	3
Coup	0.01	0.09	0	1
Population (log)	8.94	1.66	4.52	13.82
Autocratizing countries (%, global)	9.52	6.49	0.00	25.71
Democratizing countries (%, global)	19.20	8.48	0.00	41.76

Table 10. Episode sample including only episode years (n = 352 country-years)

	Mean	St. Dev.	Min	Max
Judicial constraints on executive 0.66	0.20	0.19	0.98	
Legislative constraints on executive	0.62	0.23	0.12	0.93
GDP per capita (log)	8.60	0.79	6.55	10.47
GDP growth (5 year avg.)	1.27	3.77	−21.73	9.54
Regional democracy levels	0.48	0.14	0.15	0.71
Democratic Stock 0.50	0.14	0.17	0.76	
Previous autocratization episodes	1.28	0.54	1	3
Coup	0.05	0.21	0	1
Population (log)	9.24	1.24	5.75	13.32
Autocratizing countries (%, global)	10.88	6.28	0.00	25.14
Democratizing countries (%, global)	16.67	8.27	2.00	41.76
Episode duration	4.58	3.73	0	22

E Robustness Checks

In this section, we report a series of robustness tests and additional analyses. First, we run two variants of our main models (Table 11). Model 3 is an onset model with country dummies, often referred to as country fixed-effects, instead of region dummies. The results are similar to Model 2 in the main text (Table 3), including the positive and significant effect for GDP per capita and democratic stock. However, we note one deviation from the main model: the coefficient for judicial constraints has switched signs. As stated earlier, autocratization episodes in democracies are rare events. Variation lies mainly *between* countries. In addition, judicial constraints is a variable that changes slowly. Therefore, we prefer the inclusion of region dummies over country dummies in our main analysis. Model 4 is a probit regression using only second stage observations, thus ignoring the selection process. The results are highly comparable to the second stage results from the selection models in the main table.

Second, we rerun our main models including all components of the executive constraints indices as separate predictors to test if certain sub-components are particularly influential (Table 12). Model 5 (selection) and 7 (onset) include all sub-indicators from the judicial constraints index and Model 6 and 8 all sub-indicators from the legislative constraints index. The indicators for the variables "executive respects constitution" stick out as significant predictors for onset and the high court seems to be of particular relevance for breakdown resilience. and "legislature opposition parties" stick out as significant predictors of breakdown resilience. The results for all other variables are highly comparable to our main models.

Third, our operationalization of episodes depends on several deliberate choices regarding how to identify start and end dates of episodes. While we have chosen theoretically motivated default parameters, we run additional models where we modify these (Table 13). For Model 9, we reduce the threshold to avert democratic breakdown in one year from 0.03 to 0.02 in line with Lührmann and Lindberg. In Model 11, we use an increment of 0.15 to create the episodes, Model 12 is based on the lower bounds of the EDI (codelow) and for Model 13, we use the upper bounds of the EDI credible intervals (codehigh) to generate the episodes. Overall, our main variables of interest (judicial constraints, democratic stock, and regional levels of democracy) and all other variables show similar results in these additional tests. The coefficient for judicial constraints is positive and significant except for Model 12. Democratic stock is positive and significant in every model. Regional levels of democracy, is less robust. The coefficient fails to reach statistical significance in Models 9 and 12. Overall, these robustness tests corroborate our initial results.

Table 11. Robustness checks: Onset model with country dummies and pooled probit model ignoring selection effects

	Model 3	Model 4
Judicial constraints on executive	-2.70^{***}	1.92^{*}
	(0.44)	(0.75)
Legislative constraints on executive	0.62	-0.03
	(0.33)	(0.67)
GDP per capita (log)	0.37^{*}	0.06
	(0.16)	(0.31)
GDP growth (5 year avg)	0.00	0.02
	(0.0)	(0.03)
Regional democracy levels	1.01	$4.81^{\dagger}$
	(0.70)	(1.34)
Democratic stock	3.17^{**}	3.29^{*}
	(0.60)	(1.60)
Coup	-1.05^{***}	-2.48^{***}
	(0.20)	(0.52)
Population (log)	0.64^{*}	0.10
	(0.27)	(0.12)
Previous aut. episodes	-2.28^{***}	
	(0.08)	
Autocratizing countries (%, global)	0.02^{**}	
	(0.01)	
Democratizing countries (%, global)	0.01	
	(0.00)	
Episode duration		-0.20^{**}
		(0.06)
Episode duration^2		0.01^{*}
		(0.00)
t	-0.12^{***}	
	(0.01)	
t^2	0.09^{***}	
	(0.02)	
t^3	0.00^{***}	
	(0.00)	
Intercept	-9.51^{***}	-4.20
	(3.51)	(3.92)
Nonlinear time trend	yes	yes
Region dummies	no	yes
Country dummies	yes	no
AIC	583.29	258.51
BIC	1355.79	339.64
Log Likelihood	-168.65	-108.25
Num. obs.	3,946	352

Model 3 estimates episode onset including country dummies and time since last episode onset (t). DV: democratic regression onset. Model 4 is a probit model for ongoing episodes of democratic regression without taking into account selection effects. DV: democratic breakdown. Standard errors clustered at the country-level. Significance levels $^{***}p < 0.001$, $^{**}p < 0.01$, $^{*}p < 0.05$, $^{\dagger}p < 0.10$.

Table 12. Robustness checks: Subcomponents of executive constraints indices for selection models (Model 5 and 6) and onset models (Model 7 and 8)

	Model 5		Model 6		Model 7	Model 8
	Selection	Outcome	Selection	Outcome	Onset	Onset
Executive respects constitution	−0.33*	0.25			0.45***	
	(0.17)	(0.47)			(0.11)	
Compliance with judiciary	−0.26	−0.27			0.17	
	(0.21)	(0.26)			(0.14)	
Compliance with high court	−0.13	0.57*			−0.03	
	(0.18)	(0.27)			(0.16)	
High court independence	−0.01	0.48†			−0.12	
	(0.19)	(0.27)			(0.13)	
Lower court independence	−0.12	−0.46*			0.04	
	(0.06)	(0.23)			(0.17)	
Legislature questions officials			−0.28*	−0.54*		0.16
			(0.14)	(0.17)		(0.10)
Executive oversight			0.04	0.25		−0.19
			(0.14)	(0.18)		(0.12)
Legislature investigates in practice			−0.17	−0.12		0.15
			(0.13)	(0.17)		(0.11)
Legislature opposition parties			0.15	0.43*		−0.21
			(0.15)	(0.19)		(0.12)
Judicial constraints on executive			−2.60***	2.15*		1.80**
			(0.78)	(0.98)		(0.58)
Legislative constraints on executive	−1.29*	−0.44			−0.50	
	(0.53)	(0.76)			(0.41)	
GDP per capita (log)	−1.01***	0.11	−0.96***	0.13	0.39	0.33
	(0.25)	(0.37)	(0.23)	(0.32)	(0.21)	(0.17)
GDP growth (5 year avg)	−0.04**	0.01	−0.05***	0.00	0.01	0.01
	(0.02)	(0.05)	(0.78)	(0.01)	(0.01)	(0.02)
Regional democracy levels	−0.42	3.25	0.38	4.29	0.03	−0.08
	(1.29)	(2.80)	(1.28)	(3.35)	(0.81)	(1.06)
Democratic stock	2.10†	4.00**	1.68†	3.37*	5.73***	5.56***
	(1.10)	(1.41)	(3.59)	(0.97)	(1.41)	(0.99)
Coup	1.45***	−2.37***	−1.50***	−2.68***	−1.46***	−1.41***
	(0.29)	(0.62)	(0.31)	(0.71)	(0.25)	(0.35)
Population (log)	0.02	0.20	0.07	0.20*	−0.03	−0.09
	(0.09)	(0.13)	(0.09)	(0.12)	(0.05)	(0.04)
Previous aut. episodes	1.33***		1.34***		−1.35***	−1.31***
	(0.16)		(0.15)		(0.11)	(0.05)
Autocratizing countries (%, global)	−0.01		−0.01		0.02	0.02
	(0.02)		(0.02)		(0.01)	(0.02)
Democratizing countries (%, global)	−0.03**		−0.04***		0.02*	0.02
	(0.01)		(0.01)		(0.01)	(0.01)
Episode duration		−0.24**		−0.17***		
		(0.08)		(0.05)		
Episode duration^2		0.01*		0.00		
		(0.02)		(0.02)		
Intercept	9.39***	−3.71	9.18***	−5.79	−4.34*	−4.49**
	(1.97)	(3.19)	(1.84)	(4.45)	(1.76)	(1.41)
ρ	0.26		0.49			
	(0.62)		(0.48)			
Nonlinear time trend	yes		yes		yes	yes
Region dummies (1st stage)	yes		yes		yes	yes
AIC	1514.57		1511.51		515.91	520.45
BIC	1844.60		1828.84		698.05	696.30
Log Likelihood	-705.28		-705.75		-228.95	-232.22
Total obs.	4,216		4,216		3,946	3,946
Censored obs.	3,864		3,864		–	–
Obs. in outcome stage	352		352		–	–

Components of judicial constraints as labelled in the V-Dem codebook: v2exrescon, v2jucomp, v2juhccomp, v2juhcind, v2juncind; components of legislative constraints: v2lgqstexp, v2lgotovst, v2lginvstp, v2lgoppart. Standard errors clustered at the country-level. Significance levels $^{***}p < 0.001$, $^{**}p < 0.01$, $^{*}p < 0.05$, $^{\dagger}p < 0.10$.

Table 13. Robustness checks: Different operationalization of episodes.

	Model 9		Model 10	
	Selection	Outcome	Selection	Outcome
Judicial constraints on executive	-2.12^{***}	1.90^{*}	-1.42^{**}	2.04^{*}
	(0.61)	(0.91)	(0.48)	(0.79)
Legislative constraints on executive	-1.19^{**}	-0.16	-1.20^{***}	-0.09
	(0.43)	(0.66)	(0.33)	(0.51)
GDP per capita (log)	-0.99^{**}	$-0.73^{\dagger}$	-0.44^{**}	0.11
	(0.18)	(0.37)	(0.13)	(0.22)
GDP growth (5 year avg)	-0.04^{*}	0.01	$-0.02^{\dagger}$	0.01
	(0.01)	(0.03)	(0.01)	(0.02)
Regional democracy levels	-1.10	1.93	0.31	5.77^{**}
	(1.10)	(2.96)	(1.06)	(2.02)
Democratic stock	1.64^{*}	4.29^{**}	0.93	3.51^{**}
	(0.78)	(1.31)	(0.61)	(1.18)
Coup	1.42^{***}	-1.86^{***}	1.03^{***}	-2.33^{***}
	(0.27)	(0.91)	(0.30)	(0.52)
Population (log)	0.01	0.01	0.01	0.07
	(0.06)	(0.14)	(0.01)	(0.10)
Previous aut. episodes	1.16^{***}		0.53^{***}	
	(0.16)		(0.11)	
Autocratizing countries (%, global)	-0.01		0.00	
	(0.02)		(0.01)	
Democratizing countries (%, global)	-0.04^{***}		-0.02^{**}	
	(0.01)		(0.01)	
Episode duration		-0.12		-0.22^{***}
		(0.13)		(0.06)
Episode duration^2		0.00		0.01^{**}
		(0.00)		(0.00)
Intercept	9.88^{***}	2.86	5.02^{***}	$-4.95^{\dagger}$
	(1.55)	(4.36)	(0.94)	(2.56)
ρ	-0.18		0.28	
	(0.38)		(0.64)	
Nonlinear time trend	yes		yes	
Region dummies (1st stage)	yes		yes	
AIC	1242.37		2411.79	
BIC	1567.36		2734.53	
Log Likelihood	-569.18		-1153.89	
Total obs.	4,257		3,665	
Censored obs.	3,955		3,069	
Obs. in outcome stage	302		596	

Model 9 uses a sudden turn threshold of 0.02. Model 10 uses a threshold of 0.05 change in EDI to identify manifest episodes. Dependent variable in selection equation: autocratization. Dependent variable in outcome equation: breakdown resilience. Standard errors clustered at the country-level. Significance levels $^{***}p < 0.001$, $^{**}p < 0.01$, $^{*}p < 0.05$, $^{\dagger}p < 0.10$.

Table 14. Robustness checks: Different operationalization of episodes.

	Model 11		Model 12	
	Selection	Outcome	Selection	Outcome
Judicial constraints on executive	-3.12^{***}	1.54^{*}	-2.85^{***}	-0.36
	(0.68)	(0.69)	(0.69)	(0.92)
Legislative constraints on executive	-1.62^{**}	-0.09	-0.63	0.35
	(0.52)	(0.51)	(0.53)	(0.77)
GDP per capita (log)	0.98^{**}	0.12	-0.59^{***}	-0.30
	(0.25)	(0.14)	(0.15)	(0.20)
GDP growth (5 year avg)	-0.05^{**}	0.01	-0.02	0.01
	(0.01)	(0.02)	(0.01)	(0.02)
Regional democracy levels	0.24	3.02^{***}	-0.71	3.37
	(1.62)	(0.73)	(1.02)	(1.12)
Democratic stock	$2.12^{\dagger}$	2.84^{*}	0.91	$2.73^{\dagger}$
	(1.16)	(1.14)	(0.81)	(0.94)
Coup	1.67^{***}	-2.35^{***}	0.85^{**}	-1.15^{*}
	(0.27)	(0.39)	(0.31)	(0.57)
Population (log)	0.06	0.15	0.12^{*}	0.05
	(0.01)	(0.08)	(0.07)	(0.098)
Previous aut. episodes	1.37^{***}		0.87^{***}	
	(0.12)		(0.12)	
Autocratizing countries (%, global)	0.03		-0.00	
	(0.03)		(0.01)	
Democratizing countries (%, global)	-0.04^{**}		-0.02^{**}	
	(0.01)		(0.01)	
Episode duration		-0.22^{**}		-0.02
		(0.06)		(0.067)
Episode duration^2		0.01^{**}		0.00
		(0.00)		(0.00)
Intercept	10.01^{***}	-6.92^{***}	6.86^{***}	1.72
	(1.95)	(1.69)	(1.28)	(1.92)
ρ	-0.18		0.28	
	(0.38)		(0.64)	
Nonlinear time trend	yes		yes	
Region dummies (1st stage)	yes		yes	
AIC	1242.37		2411.79	
BIC	1567.36		2734.53	
Log Likelihood	-569.18		-1153.89	
Total obs.	3,827		3,665	
Censored obs.	3,544		3,069	
Obs. in outcome stage	283		596	

Model 11 uses increments of .15 to identify manifest episodes. Model 12 uses the lower bound of the EDI to create the episodes data. Dependent variable in selection equation: autocratization. Dependent variable in outcome equation: breakdown resilience. Standard errors clustered at the country-level. Significance levels $^{***}p < 0.001$, $^{**}p < 0.01$, $^{*}p < 0.05$, $^{\dagger}p < 0.10$.

Table 15. Robustness checks: Different operationalization of episodes.

	Model 13 Selection	Outcome
Judicial constraints on executive	-1.18^{*}	1.92^{**}
	(0.82)	(0.62)
Legislative constraints on executive	-1.43^{***}	0.07
	(0.41)	(0.66)
GDP per capita (log)	-0.52^{**}	0.01
	(0.19)	(0.24)
GDP growth (5 year avg)	-0.01	0.07^{**}
	(0.01)	(0.02)
Regional democracy levels	0.48	2.24^{**}
	(1.19)	(2.22)
Democratic stock	1.03	2.77^{**}
	(0.83)	(0.90)
Coup	1.33^{***}	-2.29^{***}
	(0.24)	(0.45)
Population (log)	0.06	0.08
	(0.05)	(0.06)
Previous aut. episodes	0.86^{***}	
	(0.12)	
Autocratizing countries (%, global)	0.01	
	(0.01)	
Democratizing countries (%, global)	-0.02^{**}	
	(0.01)	
Episode duration		-0.16
		(0.13)
Episode duration^2		0.00
		(0.01)
Intercept	4.91^{**}	$-4.14^{\dagger}$
	(1.71)	(2.17)
ρ	-0.18	
	(0.38)	
Nonlinear time trend	yes	
Region dummies (1st stage)	yes	
AIC	1242.37	
BIC	1567.36	
Log Likelihood	-569.18	
Total obs.	3,827	
Censored obs.	3,544	
Obs. in outcome stage	283	

Model 13 uses the upper bound of the EDI to create the episodes data. Dependent variable in selection equation: autocratization. Dependent variable in outcome equation: democratic breakdown. Standard errors clustered at the country-level. Significance levels $^{***}p < 0.001$, $^{**}p < 0.01$, $^{*}p < 0.05$, $^{\dagger}p < 0.10$.
